# 2-[1-Chloro-3-(2-methyl-5-nitro-1*H*-imidazol-1-yl)propan-2-yloxycarbonyl]benzoic acid

**DOI:** 10.1107/S1600536808001268

**Published:** 2008-01-18

**Authors:** Xiao Tao, Lin Yuan, Xiao-Qing Zhang, Jin-Tang Wang

**Affiliations:** a Department of Applied Chemistry, College of Science, Nanjing University of Technology, Nanjing 210009, People’s Republic of China; b Nanjing Huawei Medicinal Science Development Co. Ltd, Nanjing 210036, People’s Republic of China

## Abstract

The asymmetric unit of the title compound, C_15_H_14_ClN_3_O_6_, contains two independent mol­ecules. The imidazole rings are oriented with respect to the benzene rings at dihedral angles of 19.66 (3) and 21.64 (3)°. In the crystal structure, inter­molecular O—H⋯N hydrogen bonds link the mol­ecules into infinite chains.

## Related literature

For bond-length data, see: Allen *et al.* (1987 ▶).
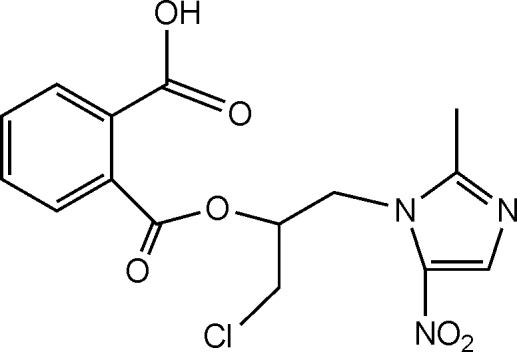


## Experimental

### 

#### Crystal data

C_15_H_14_ClN_3_O_6_
*M* *_r_* = 367.74
Monoclinic, 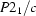

*a* = 15.214 (3) Å
*b* = 16.271 (3) Å
*c* = 15.069 (3) Å
β = 113.86 (3)°
*V* = 3411.5 (14) Å^3^
*Z* = 8
Mo *K*α radiation
μ = 0.26 mm^−1^
*T* = 294 (2) K
0.40 × 0.30 × 0.20 mm

#### Data collection

Enraf–Nonius CAD-4 diffractometer
Absorption correction: ψ scan (North *et al.*, 1968 ▶) *T*_min_ = 0.903, *T*_max_ = 0.950
6938 measured reflections
6682 independent reflections
3559 reflections with *I* > 2σ(*I*)
*R*_int_ = 0.037
3 standard reflections frequency: 120 min intensity decay: none

#### Refinement

*R*[*F* ^2^ > 2σ(*F* ^2^)] = 0.066
*wR*(*F* ^2^) = 0.175
*S* = 1.02
6682 reflections
451 parameters
H-atom parameters constrained
Δρ_max_ = 0.41 e Å^−3^
Δρ_min_ = −0.36 e Å^−3^

### 

Data collection: *CAD-4 Software* (Enraf–Nonius, 1989 ▶); cell refinement: *CAD-4 Software*; data reduction: *XCAD4* (Harms & Wocadlo, 1995 ▶); program(s) used to solve structure: *SHELXS97* (Sheldrick, 2008 ▶); program(s) used to refine structure: *SHELXL97* (Sheldrick, 2008 ▶); molecular graphics: *SHELXTL* (Sheldrick, 2008 ▶); software used to prepare material for publication: *SHELXTL*.

## Supplementary Material

Crystal structure: contains datablocks I, global. DOI: 10.1107/S1600536808001268/hk2413sup1.cif

Structure factors: contains datablocks I. DOI: 10.1107/S1600536808001268/hk2413Isup2.hkl

Additional supplementary materials:  crystallographic information; 3D view; checkCIF report

## Figures and Tables

**Table 1:** Hydrogen-bond geometry (Å, °)

*D*—H⋯*A*	*D*—H	H⋯*A*	*D*⋯*A*	*D*—H⋯*A*
O1—H1*B*⋯N5^i^	0.82	1.81	2.623 (3)	172
O7—H7*A*⋯N2^ii^	0.82	1.82	2.621 (3)	166

Symmetry codes: (i) 
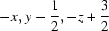
; (ii) 
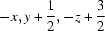
.

**Fig. 1.**
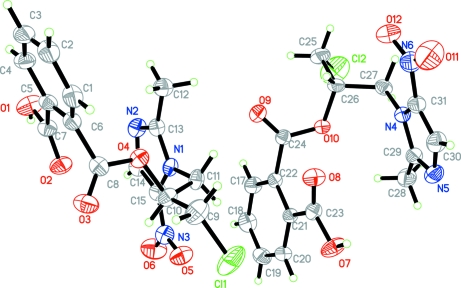
Ellipsoid plot.

**Fig. 2.**
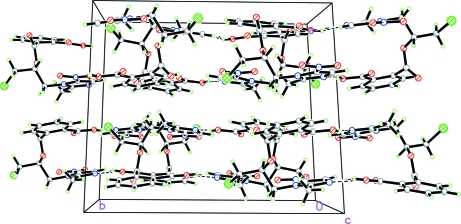
Packing diagram.

## supplementary crystallographic information

### Comment

As part of our ongoing studies, we synthesized the title compound, (I), and report herein its crystal structure.

The asymmetric unit of the title compound, (I), contains two independent molecules (Fig. 1), in which the bond lengths are within normal ranges (Allen *et al.*, 1987).

Rings A (C1–C6), B (N1/N2/C13–C15), C (C17–C22) and D (N4/N5/C29–C31) are, of course, planar and the dihedral angles between them are A/B = 19.66 (3)° and C/D = 21.64 (3)°.

In the crystal structure, intermolecular O—H···N hydrogen bonds (Table 1) link the molecules into infinite chains (Fig. 2), in which they may be effective in the stabilization of the structure.

### Experimental

For the preparation of the title conpound, ornidazole (14.6 g, 66 mmol), phthalic anhydride (11.8 g, 80 mmol), acetone (80 ml) and pyridine (6 ml) were added into a three-necked round-bottom flask (250 ml) fitted with a mechanical stirrer and a reflux condensing tube. The mixture was stirred until the solids were completely dissolved, and heated to reflux for about 7 h, and then the reaction was stopped and the mixture was cooled. After filtration of the mixture under vacuum, the colorless deposition was obtained (yield; 18 g, 74%). Suitable crystals for X-ray analysis were obtained by dissolving the title compound (0.1 g) in dry methanol (5 ml), and then allowing the solution to evaporate slowly at room temperature for about 12 d.

### Refinement

H atoms were positioned geometrically, with O00—H = 0.82 Å (for OH), C-00H = 0.93, 0.98, 0.97 and 0.96 Å for aromatic, methine, methylene and methyl H, and constrained to ride on their parent atoms, with *U*_iso_(H) = *xU*_eq_(C,*O*), where *x* = 1.5 for OH and methyl H, and *x* = 1.2 for all other H atoms.

### Crystal data

**Table:**

C_15_H_14_ClN_3_O_6_	*F*_000_ = 1520
*M**_r_* = 367.74	*D*_x_ = 1.432 Mg m^−^^3^
Monoclinic, *P*2_1_/*c*	Melting point = 444–447 K
Hall symbol: -P 2ybc	Mo *K*α radiation λ = 0.71073 Å
*a* = 15.214 (3) Å	Cell parameters from 25 reflections
*b* = 16.271 (3) Å	θ = 10–13º
*c* = 15.069 (3) Å	µ = 0.26 mm^−^^1^
β = 113.86 (3)º	*T* = 294 (2) K
*V* = 3411.5 (14) Å^3^	Block, colourless
*Z* = 8	0.40 × 0.30 × 0.20 mm

### Data collection

**Table:**

Enraf–Nonius CAD-4 diffractometer	*R*_int_ = 0.037
Radiation source: fine-focus sealed tube	θ_max_ = 26.0º
Monochromator: graphite	θ_min_ = 1.5º
*T* = 294(2) K	*h* = −18→0
ω/2θ scans	*k* = 0→20
Absorption correction: ψ scan(North *et al.*, 1968)	*l* = −16→18
*T*_min_ = 0.903, *T*_max_ = 0.950	3 standard reflections
6938 measured reflections	every 120 min
6682 independent reflections	intensity decay: none
3559 reflections with *I* > 2σ(*I*)	

### Refinement

**Table:**

Refinement on *F*^2^	Secondary atom site location: difference Fourier map
Least-squares matrix: full	Hydrogen site location: inferred from neighbouring sites
*R*[*F*^2^ > 2σ(*F*^2^)] = 0.066	H-atom parameters constrained
*wR*(*F*^2^) = 0.175	*w* = 1/[σ^2^(*F*_o_^2^) + (0.07*P*)^2^ + 1.25*P*] where *P* = (*F*_o_^2^ + 2*F*_c_^2^)/3
*S* = 1.02	(Δ/σ)_max_ < 0.001
6682 reflections	Δρ_max_ = 0.41 e Å^−^^3^
451 parameters	Δρ_min_ = −0.36 e Å^−^^3^
Primary atom site location: structure-invariant direct methods	Extinction correction: none

### Special details

**Table:**

Geometry. All e.s.d.'s (except the e.s.d. in the dihedral angle between two l.s. planes) are estimated using the full covariance matrix. The cell e.s.d.'s are taken into account individually in the estimation of e.s.d.'s in distances, angles and torsion angles; correlations between e.s.d.'s in cell parameters are only used when they are defined by crystal symmetry. An approximate (isotropic) treatment of cell e.s.d.'s is used for estimating e.s.d.'s involving l.s. planes.
Refinement. Refinement of *F*^2^ against ALL reflections. The weighted *R*-factor *wR* and goodness of fit *S* are based on *F*^2^, conventional *R*-factors *R* are based on *F*, with *F* set to zero for negative *F*^2^. The threshold expression of *F*^2^ > σ(*F*^2^) is used only for calculating *R*-factors(gt) *etc*. and is not relevant to the choice of reflections for refinement. *R*-factors based on *F*^2^ are statistically about twice as large as those based on *F*, and *R*- factors based on ALL data will be even larger.

### Fractional atomic coordinates and isotropic or equivalent isotropic displacement parameters (Å^2^)

**Table:**

	*x*	*y*	*z*	*U*_iso_*/*U*_eq_	
Cl1	0.11705 (11)	0.43257 (8)	0.72492 (11)	0.1116 (6)	
Cl2	−0.38901 (14)	0.04807 (9)	0.66545 (14)	0.1333 (7)	
O1	0.3761 (2)	0.03250 (17)	1.0499 (2)	0.0784 (9)	
H1B	0.3710	0.0047	1.0026	0.118*	
O2	0.3562 (2)	0.13615 (16)	0.94718 (18)	0.0613 (7)	
O3	0.3744 (2)	0.32764 (19)	0.96664 (19)	0.0684 (8)	
O4	0.23091 (17)	0.27469 (14)	0.94386 (16)	0.0485 (6)	
O5	0.1328 (2)	0.2233 (2)	0.6314 (2)	0.0748 (8)	
O6	0.1459 (2)	0.0990 (2)	0.5886 (2)	0.0887 (10)	
O7	−0.1465 (2)	0.39839 (15)	0.55531 (19)	0.0719 (9)	
H7A	−0.1519	0.4404	0.5828	0.108*	
O8	−0.1401 (2)	0.33919 (14)	0.69075 (19)	0.0596 (7)	
O9	−0.11021 (18)	0.16192 (17)	0.77884 (18)	0.0592 (7)	
O10	−0.25765 (16)	0.19193 (14)	0.66714 (15)	0.0453 (6)	
O11	−0.3413 (3)	0.4576 (2)	0.8743 (2)	0.1134 (14)	
O12	−0.3404 (2)	0.3246 (2)	0.8740 (2)	0.0832 (10)	
N1	0.11477 (19)	0.16366 (16)	0.7989 (2)	0.0426 (7)	
N2	0.1320 (2)	0.03336 (18)	0.8448 (2)	0.0554 (8)	
N3	0.1364 (2)	0.1490 (2)	0.6445 (2)	0.0592 (9)	
N4	−0.37734 (19)	0.32555 (17)	0.6748 (2)	0.0438 (7)	
N5	−0.3757 (2)	0.4369 (2)	0.5904 (2)	0.0597 (9)	
N6	−0.3466 (3)	0.3912 (3)	0.8340 (2)	0.0684 (10)	
C1	0.3666 (3)	0.3017 (3)	1.1689 (3)	0.0580 (11)	
H1A	0.3550	0.3577	1.1582	0.070*	
C2	0.3935 (3)	0.2701 (3)	1.2607 (3)	0.0673 (13)	
H2B	0.3988	0.3047	1.3117	0.081*	
C3	0.4129 (3)	0.1871 (3)	1.2777 (3)	0.0676 (12)	
H3A	0.4320	0.1663	1.3402	0.081*	
C4	0.4037 (3)	0.1351 (3)	1.2018 (3)	0.0580 (11)	
H4A	0.4166	0.0793	1.2133	0.070*	
C5	0.3755 (2)	0.1661 (2)	1.1088 (2)	0.0464 (9)	
C6	0.3568 (2)	0.2497 (2)	1.0920 (2)	0.0462 (9)	
C7	0.3675 (3)	0.1105 (2)	1.0262 (3)	0.0498 (9)	
C8	0.3245 (3)	0.2879 (2)	0.9941 (3)	0.0475 (9)	
C9	0.1664 (3)	0.3935 (2)	0.8436 (3)	0.0749 (13)	
H9A	0.2255	0.4225	0.8812	0.090*	
H9B	0.1219	0.4027	0.8740	0.090*	
C10	0.1872 (3)	0.3018 (2)	0.8441 (2)	0.0476 (9)	
H10A	0.2298	0.2909	0.8110	0.057*	
C11	0.0955 (2)	0.2518 (2)	0.7990 (3)	0.0491 (9)	
H11A	0.0560	0.2615	0.8349	0.059*	
H11B	0.0596	0.2701	0.7328	0.059*	
C12	0.1003 (3)	0.1285 (3)	0.9559 (3)	0.0616 (11)	
H12A	0.1046	0.0788	0.9918	0.092*	
H12B	0.0378	0.1523	0.9380	0.092*	
H12C	0.1484	0.1666	0.9954	0.092*	
C13	0.1158 (2)	0.1095 (2)	0.8677 (3)	0.0465 (9)	
C14	0.1408 (3)	0.0381 (2)	0.7593 (3)	0.0569 (10)	
H14A	0.1517	−0.0061	0.7259	0.068*	
C15	0.1311 (3)	0.1176 (2)	0.7298 (3)	0.0477 (9)	
C17	−0.1188 (3)	0.1073 (2)	0.5763 (3)	0.0552 (10)	
H17A	−0.1214	0.0583	0.6069	0.066*	
C18	−0.0988 (3)	0.1060 (3)	0.4947 (3)	0.0643 (11)	
H18A	−0.0887	0.0560	0.4703	0.077*	
C19	−0.0939 (3)	0.1783 (3)	0.4494 (3)	0.0594 (11)	
H19A	−0.0811	0.1773	0.3940	0.071*	
C20	−0.1079 (2)	0.2521 (2)	0.4867 (2)	0.0477 (9)	
H20A	−0.1032	0.3009	0.4568	0.057*	
C21	−0.1288 (2)	0.2548 (2)	0.5676 (2)	0.0389 (8)	
C22	−0.1352 (2)	0.1814 (2)	0.6130 (2)	0.0399 (8)	
C23	−0.1395 (3)	0.3347 (2)	0.6110 (3)	0.0459 (9)	
C24	−0.1631 (3)	0.1788 (2)	0.6968 (3)	0.0428 (8)	
C25	−0.3119 (3)	0.1052 (3)	0.7666 (3)	0.0737 (13)	
H25A	−0.3391	0.1072	0.8147	0.088*	
H25B	−0.2503	0.0777	0.7955	0.088*	
C26	−0.2968 (3)	0.1925 (2)	0.7394 (3)	0.0491 (9)	
H26A	−0.2528	0.2216	0.7972	0.059*	
C27	−0.3904 (2)	0.2392 (2)	0.6947 (3)	0.0490 (9)	
H27A	−0.4314	0.2125	0.6345	0.059*	
H27B	−0.4229	0.2368	0.7384	0.059*	
C28	−0.4060 (3)	0.3050 (3)	0.5004 (3)	0.0655 (11)	
H28A	−0.4093	0.3398	0.4476	0.098*	
H28B	−0.4660	0.2767	0.4832	0.098*	
H28C	−0.3553	0.2656	0.5136	0.098*	
C29	−0.3866 (3)	0.3556 (2)	0.5877 (3)	0.0494 (9)	
C30	−0.3610 (3)	0.4604 (3)	0.6814 (3)	0.0618 (11)	
H30A	−0.3517	0.5142	0.7039	0.074*	
C31	−0.3617 (3)	0.3934 (2)	0.7348 (3)	0.0508 (9)	

### Atomic displacement parameters (Å^2^)

**Table:**

	*U*^11^	*U*^22^	*U*^33^	*U*^12^	*U*^13^	*U*^23^
Cl1	0.1335 (12)	0.0557 (8)	0.1106 (11)	0.0032 (8)	0.0131 (9)	0.0320 (7)
Cl2	0.1632 (15)	0.0625 (9)	0.1512 (15)	−0.0410 (9)	0.0400 (12)	0.0081 (9)
O1	0.128 (3)	0.0514 (19)	0.0574 (18)	−0.0074 (17)	0.0396 (18)	−0.0063 (14)
O2	0.085 (2)	0.0620 (18)	0.0400 (15)	0.0030 (15)	0.0285 (14)	−0.0037 (13)
O3	0.0697 (19)	0.086 (2)	0.0548 (17)	−0.0269 (16)	0.0310 (15)	−0.0091 (15)
O4	0.0524 (16)	0.0461 (14)	0.0426 (14)	−0.0039 (12)	0.0146 (12)	−0.0052 (11)
O5	0.099 (2)	0.060 (2)	0.0677 (19)	−0.0005 (17)	0.0358 (17)	0.0091 (16)
O6	0.124 (3)	0.083 (2)	0.078 (2)	−0.011 (2)	0.059 (2)	−0.0271 (19)
O7	0.126 (3)	0.0319 (15)	0.0609 (17)	0.0040 (15)	0.0406 (17)	0.0059 (13)
O8	0.093 (2)	0.0387 (15)	0.0592 (17)	−0.0026 (13)	0.0427 (16)	−0.0068 (13)
O9	0.0560 (16)	0.0704 (19)	0.0476 (16)	0.0104 (14)	0.0173 (13)	0.0095 (14)
O10	0.0485 (15)	0.0482 (14)	0.0430 (13)	0.0016 (12)	0.0225 (12)	0.0073 (11)
O11	0.165 (4)	0.093 (3)	0.071 (2)	0.024 (3)	0.037 (2)	−0.026 (2)
O12	0.098 (2)	0.098 (3)	0.0586 (19)	0.004 (2)	0.0372 (18)	0.0143 (18)
N1	0.0440 (17)	0.0327 (16)	0.0447 (17)	0.0032 (13)	0.0115 (13)	−0.0036 (14)
N2	0.066 (2)	0.0311 (17)	0.066 (2)	−0.0015 (15)	0.0234 (17)	−0.0005 (15)
N3	0.061 (2)	0.056 (2)	0.056 (2)	−0.0040 (18)	0.0196 (17)	−0.0071 (19)
N4	0.0451 (17)	0.0426 (18)	0.0454 (17)	0.0005 (14)	0.0199 (14)	0.0039 (14)
N5	0.067 (2)	0.050 (2)	0.061 (2)	0.0025 (17)	0.0253 (18)	0.0116 (17)
N6	0.071 (2)	0.082 (3)	0.049 (2)	0.007 (2)	0.0225 (18)	−0.006 (2)
C1	0.053 (2)	0.070 (3)	0.049 (2)	0.003 (2)	0.0197 (19)	−0.023 (2)
C2	0.056 (3)	0.103 (4)	0.042 (2)	−0.002 (3)	0.0194 (19)	−0.029 (2)
C3	0.058 (3)	0.109 (4)	0.037 (2)	−0.006 (3)	0.0205 (19)	−0.007 (2)
C4	0.062 (3)	0.073 (3)	0.042 (2)	−0.013 (2)	0.0236 (19)	−0.003 (2)
C5	0.043 (2)	0.060 (3)	0.038 (2)	−0.0115 (18)	0.0194 (16)	−0.0109 (18)
C6	0.042 (2)	0.057 (2)	0.040 (2)	−0.0054 (18)	0.0172 (16)	−0.0101 (18)
C7	0.057 (2)	0.052 (2)	0.043 (2)	−0.0065 (19)	0.0218 (18)	−0.0092 (19)
C8	0.051 (2)	0.047 (2)	0.047 (2)	−0.0061 (18)	0.0213 (19)	−0.0142 (18)
C9	0.098 (3)	0.039 (2)	0.080 (3)	0.005 (2)	0.027 (3)	−0.001 (2)
C10	0.061 (2)	0.0322 (19)	0.047 (2)	0.0016 (17)	0.0196 (19)	−0.0002 (16)
C11	0.047 (2)	0.034 (2)	0.058 (2)	0.0078 (16)	0.0130 (18)	−0.0016 (17)
C12	0.068 (3)	0.061 (3)	0.059 (3)	0.001 (2)	0.029 (2)	0.001 (2)
C13	0.046 (2)	0.040 (2)	0.052 (2)	−0.0008 (17)	0.0182 (18)	−0.0039 (18)
C14	0.066 (3)	0.040 (2)	0.063 (3)	−0.0031 (19)	0.025 (2)	−0.0110 (19)
C15	0.053 (2)	0.036 (2)	0.052 (2)	−0.0026 (17)	0.0184 (18)	−0.0069 (18)
C17	0.071 (3)	0.035 (2)	0.063 (3)	0.0029 (19)	0.031 (2)	−0.0004 (18)
C18	0.077 (3)	0.047 (2)	0.072 (3)	0.003 (2)	0.033 (2)	−0.019 (2)
C19	0.064 (3)	0.069 (3)	0.055 (2)	−0.001 (2)	0.033 (2)	−0.010 (2)
C20	0.054 (2)	0.048 (2)	0.045 (2)	−0.0027 (18)	0.0246 (18)	0.0026 (17)
C21	0.0421 (19)	0.0359 (19)	0.0389 (19)	0.0000 (16)	0.0165 (16)	0.0000 (15)
C22	0.041 (2)	0.0353 (19)	0.044 (2)	−0.0007 (16)	0.0175 (16)	−0.0013 (15)
C23	0.052 (2)	0.034 (2)	0.051 (2)	−0.0003 (16)	0.0193 (18)	−0.0002 (17)
C24	0.048 (2)	0.0344 (19)	0.047 (2)	−0.0010 (17)	0.0209 (18)	0.0019 (16)
C25	0.091 (3)	0.064 (3)	0.079 (3)	0.007 (3)	0.048 (3)	0.028 (2)
C26	0.056 (2)	0.050 (2)	0.050 (2)	−0.0022 (18)	0.0307 (19)	0.0088 (18)
C27	0.049 (2)	0.047 (2)	0.057 (2)	−0.0090 (18)	0.0280 (19)	0.0007 (18)
C28	0.074 (3)	0.073 (3)	0.047 (2)	0.008 (2)	0.022 (2)	−0.002 (2)
C29	0.046 (2)	0.059 (3)	0.042 (2)	0.0018 (19)	0.0163 (17)	0.0036 (18)
C30	0.070 (3)	0.045 (2)	0.071 (3)	0.001 (2)	0.028 (2)	0.000 (2)
C31	0.054 (2)	0.055 (2)	0.044 (2)	0.0006 (19)	0.0203 (18)	−0.0033 (19)

### Geometric parameters (Å, °)

**Table:**

Cl1—C9	1.755 (4)	C9—C10	1.524 (5)
Cl2—C25	1.765 (5)	C9—H9A	0.9700
O1—C7	1.311 (4)	C9—H9B	0.9700
O1—H1B	0.8200	C10—C11	1.517 (5)
O2—C7	1.207 (4)	C10—H10A	0.9800
O3—C8	1.191 (4)	C11—H11A	0.9700
O4—C8	1.333 (4)	C11—H11B	0.9700
O4—C10	1.446 (4)	C12—C13	1.474 (5)
O5—N3	1.222 (4)	C12—H12A	0.9600
O6—N3	1.221 (4)	C12—H12B	0.9600
O7—C23	1.310 (4)	C12—H12C	0.9600
O7—H7A	0.8200	C14—C15	1.357 (5)
O8—C23	1.207 (4)	C14—H14A	0.9300
O9—C24	1.203 (4)	C15—N3	1.416 (5)
O10—C24	1.340 (4)	C17—C18	1.381 (5)
O10—C26	1.436 (4)	C17—C22	1.390 (5)
O11—N6	1.226 (5)	C17—H17A	0.9300
O12—N6	1.225 (4)	C18—C19	1.378 (6)
N1—C13	1.356 (4)	C18—H18A	0.9300
N1—C15	1.383 (4)	C19—C20	1.379 (5)
N1—C11	1.465 (4)	C19—H19A	0.9300
N2—C13	1.336 (4)	C20—C21	1.379 (5)
N2—C14	1.351 (5)	C20—H20A	0.9300
N4—C29	1.353 (4)	C21—C22	1.398 (4)
N4—C31	1.385 (4)	C21—C23	1.494 (5)
N4—C27	1.468 (4)	C22—C24	1.486 (5)
N5—C29	1.331 (5)	C25—C26	1.522 (5)
N5—C30	1.351 (5)	C25—H25A	0.9700
C1—C2	1.373 (6)	C25—H25B	0.9700
C1—C6	1.394 (5)	C26—C27	1.511 (5)
C1—H1A	0.9300	C26—H26A	0.9800
C2—C3	1.384 (6)	C27—H27A	0.9700
C2—H2B	0.9300	C27—H27B	0.9700
C3—C4	1.384 (5)	C28—C29	1.478 (5)
C3—H3A	0.9300	C28—H28A	0.9600
C4—C5	1.383 (5)	C28—H28B	0.9600
C4—H4A	0.9300	C28—H28C	0.9600
C5—C6	1.392 (5)	C30—C31	1.357 (5)
C5—C7	1.503 (5)	C30—H30A	0.9300
C6—C8	1.489 (5)	C31—N6	1.419 (5)
			
C7—O1—H1B	109.5	N2—C13—N1	110.6 (3)
C8—O4—C10	118.6 (3)	N2—C13—C12	122.7 (3)
C23—O7—H7A	109.5	N1—C13—C12	126.7 (3)
C24—O10—C26	117.8 (3)	N2—C14—C15	109.1 (3)
C13—N1—C15	105.9 (3)	N2—C14—H14A	125.5
C13—N1—C11	125.1 (3)	C15—C14—H14A	125.5
C15—N1—C11	128.9 (3)	C14—C15—N1	107.4 (3)
C13—N2—C14	107.0 (3)	C14—C15—N3	127.0 (3)
O6—N3—O5	123.8 (4)	N1—C15—N3	125.6 (3)
O6—N3—C15	116.9 (3)	C18—C17—C22	120.5 (4)
O5—N3—C15	119.2 (3)	C18—C17—H17A	119.8
C29—N4—C31	105.5 (3)	C22—C17—H17A	119.8
C29—N4—C27	125.1 (3)	C19—C18—C17	120.3 (4)
C31—N4—C27	129.1 (3)	C19—C18—H18A	119.9
C29—N5—C30	106.3 (3)	C17—C18—H18A	119.9
O12—N6—O11	124.0 (4)	C18—C19—C20	119.5 (3)
O12—N6—C31	119.2 (4)	C18—C19—H19A	120.2
O11—N6—C31	116.8 (4)	C20—C19—H19A	120.2
C2—C1—C6	119.9 (4)	C19—C20—C21	121.1 (3)
C2—C1—H1A	120.1	C19—C20—H20A	119.4
C6—C1—H1A	120.1	C21—C20—H20A	119.4
C1—C2—C3	120.4 (4)	C20—C21—C22	119.5 (3)
C1—C2—H2B	119.8	C20—C21—C23	121.3 (3)
C3—C2—H2B	119.8	C22—C21—C23	119.2 (3)
C4—C3—C2	120.0 (4)	C17—C22—C21	119.1 (3)
C4—C3—H3A	120.0	C17—C22—C24	118.1 (3)
C2—C3—H3A	120.0	C21—C22—C24	122.7 (3)
C5—C4—C3	120.0 (4)	O8—C23—O7	123.9 (3)
C5—C4—H4A	120.0	O8—C23—C21	122.3 (3)
C3—C4—H4A	120.0	O7—C23—C21	113.8 (3)
C4—C5—C6	119.8 (3)	O9—C24—O10	124.1 (3)
C4—C5—C7	120.5 (4)	O9—C24—C22	125.3 (3)
C6—C5—C7	119.7 (3)	O10—C24—C22	110.4 (3)
C5—C6—C1	119.8 (3)	C26—C25—Cl2	112.3 (3)
C5—C6—C8	123.3 (3)	C26—C25—H25A	109.1
C1—C6—C8	116.9 (4)	Cl2—C25—H25A	109.1
O2—C7—O1	124.2 (3)	C26—C25—H25B	109.1
O2—C7—C5	122.8 (4)	Cl2—C25—H25B	109.1
O1—C7—C5	113.0 (3)	H25A—C25—H25B	107.9
O3—C8—O4	125.1 (4)	O10—C26—C27	105.7 (3)
O3—C8—C6	124.7 (3)	O10—C26—C25	110.6 (3)
O4—C8—C6	110.1 (3)	C27—C26—C25	111.7 (3)
C10—C9—Cl1	111.2 (3)	O10—C26—H26A	109.6
C10—C9—H9A	109.4	C27—C26—H26A	109.6
Cl1—C9—H9A	109.4	C25—C26—H26A	109.6
C10—C9—H9B	109.4	N4—C27—C26	113.0 (3)
Cl1—C9—H9B	109.4	N4—C27—H27A	109.0
H9A—C9—H9B	108.0	C26—C27—H27A	109.0
O4—C10—C11	104.8 (3)	N4—C27—H27B	109.0
O4—C10—C9	108.1 (3)	C26—C27—H27B	109.0
C11—C10—C9	111.8 (3)	H27A—C27—H27B	107.8
O4—C10—H10A	110.7	C29—C28—H28A	109.5
C11—C10—H10A	110.7	C29—C28—H28B	109.5
C9—C10—H10A	110.7	H28A—C28—H28B	109.5
N1—C11—C10	112.1 (3)	C29—C28—H28C	109.5
N1—C11—H11A	109.2	H28A—C28—H28C	109.5
C10—C11—H11A	109.2	H28B—C28—H28C	109.5
N1—C11—H11B	109.2	N5—C29—N4	111.5 (3)
C10—C11—H11B	109.2	N5—C29—C28	124.0 (4)
H11A—C11—H11B	107.9	N4—C29—C28	124.5 (4)
C13—C12—H12A	109.5	N5—C30—C31	109.7 (4)
C13—C12—H12B	109.5	N5—C30—H30A	125.2
H12A—C12—H12B	109.5	C31—C30—H30A	125.2
C13—C12—H12C	109.5	C30—C31—N4	107.1 (3)
H12A—C12—H12C	109.5	C30—C31—N6	127.4 (4)
H12B—C12—H12C	109.5	N4—C31—N6	125.5 (4)
			
C10—O4—C8—O3	7.7 (5)	C6—C5—C7—O2	8.8 (5)
C10—O4—C8—C6	−175.5 (3)	C4—C5—C7—O1	9.1 (5)
C8—O4—C10—C11	159.8 (3)	C6—C5—C7—O1	−172.6 (3)
C8—O4—C10—C9	−80.9 (4)	C5—C6—C8—O3	−104.2 (5)
C26—O10—C24—O9	5.3 (5)	C1—C6—C8—O3	76.8 (5)
C26—O10—C24—C22	−179.2 (3)	C5—C6—C8—O4	78.9 (4)
C24—O10—C26—C27	158.9 (3)	C1—C6—C8—O4	−100.0 (4)
C24—O10—C26—C25	−80.0 (4)	Cl1—C9—C10—O4	177.8 (3)
C13—N1—C11—C10	94.9 (4)	Cl1—C9—C10—C11	−67.4 (4)
C15—N1—C11—C10	−88.2 (4)	O4—C10—C11—N1	−63.4 (4)
C15—N1—C13—N2	0.1 (4)	C9—C10—C11—N1	179.8 (3)
C11—N1—C13—N2	177.6 (3)	N2—C14—C15—N1	−0.6 (4)
C15—N1—C13—C12	−179.0 (3)	N2—C14—C15—N3	179.4 (3)
C11—N1—C13—C12	−1.5 (6)	C14—C15—N3—O6	4.0 (6)
C13—N1—C15—C14	0.3 (4)	N1—C15—N3—O6	−176.0 (3)
C11—N1—C15—C14	−177.1 (3)	C14—C15—N3—O5	−174.9 (4)
C13—N1—C15—N3	−179.7 (3)	N1—C15—N3—O5	5.1 (6)
C11—N1—C15—N3	3.0 (6)	C22—C17—C18—C19	0.7 (6)
C14—N2—C13—N1	−0.5 (4)	C18—C17—C22—C21	−1.6 (5)
C14—N2—C13—C12	178.7 (3)	C18—C17—C22—C24	175.8 (3)
C13—N2—C14—C15	0.7 (4)	C17—C18—C19—C20	0.8 (6)
C29—N4—C27—C26	97.5 (4)	C18—C19—C20—C21	−1.3 (6)
C31—N4—C27—C26	−88.5 (4)	C19—C20—C21—C22	0.4 (5)
C31—N4—C29—N5	1.3 (4)	C19—C20—C21—C23	177.1 (3)
C27—N4—C29—N5	176.5 (3)	C20—C21—C22—C17	1.0 (5)
C31—N4—C29—C28	−178.8 (3)	C23—C21—C22—C17	−175.7 (3)
C27—N4—C29—C28	−3.6 (5)	C20—C21—C22—C24	−176.3 (3)
C29—N4—C31—C30	−0.9 (4)	C23—C21—C22—C24	7.0 (5)
C27—N4—C31—C30	−175.8 (3)	C20—C21—C23—O8	−166.0 (3)
C29—N4—C31—N6	−179.1 (3)	C22—C21—C23—O8	10.7 (5)
C27—N4—C31—N6	5.9 (6)	C20—C21—C23—O7	12.9 (5)
C30—N5—C29—N4	−1.3 (4)	C22—C21—C23—O7	−170.4 (3)
C30—N5—C29—C28	178.9 (4)	C17—C22—C24—O9	72.7 (5)
C29—N5—C30—C31	0.7 (4)	C21—C22—C24—O9	−110.0 (4)
C6—C1—C2—C3	1.3 (6)	C17—C22—C24—O10	−102.8 (4)
C2—C1—C6—C5	−0.8 (5)	C21—C22—C24—O10	74.6 (4)
C2—C1—C6—C8	178.2 (3)	Cl2—C25—C26—O10	−58.1 (4)
C1—C2—C3—C4	−1.0 (6)	Cl2—C25—C26—C27	59.4 (4)
C2—C3—C4—C5	0.2 (6)	O10—C26—C27—N4	−64.1 (4)
C3—C4—C5—C6	0.3 (5)	C25—C26—C27—N4	175.5 (3)
C3—C4—C5—C7	178.6 (3)	N5—C30—C31—N4	0.1 (4)
C4—C5—C6—C1	0.0 (5)	N5—C30—C31—N6	178.3 (3)
C7—C5—C6—C1	−178.3 (3)	C30—C31—N6—O12	−172.9 (4)
C4—C5—C6—C8	−178.9 (3)	N4—C31—N6—O12	5.0 (6)
C7—C5—C6—C8	2.8 (5)	C30—C31—N6—O11	7.5 (6)
C4—C5—C7—O2	−169.5 (4)	N4—C31—N6—O11	−174.5 (4)

### Hydrogen-bond geometry (Å, °)

**Table:**

*D*—H···*A*	*D*—H	H···*A*	*D*···*A*	*D*—H···*A*
O1—H1B···N5^i^	0.82	1.81	2.623 (3)	172
O7—H7A···N2^ii^	0.82	1.82	2.621 (3)	166

Symmetry codes: (i) −*x*, *y*−1/2, −*z*+3/2; (ii) −*x*, *y*+1/2, −*z*+3/2.
